# Task-free functional connectivity changes before and after hyper- and hypoglycemia in very preterm neonates

**DOI:** 10.1117/1.NPh.13.S1.S13008

**Published:** 2026-03-31

**Authors:** Guy A. Perkins, Giacomo Bianco, Silvia Guiducci, Elisa De Pietri, Giulia Res, Federica Savio, Daniele Trevisanuto, Elena Priante, Eugenio Baraldi, Alfonso Galderisi, Sabrina Brigadoi

**Affiliations:** a University of Padova, Department of Developmental Psychology and Socialization, Padua, Italy; b University of Padova, Department of Information Engineering, Padua, Italy; c University of Padova, Department of Women’s and Children’s Health, Padua, Italy; d Istituto Ricerca Pediatrica “Città’ della Speranza, Padua, Italy; e Yale University, Yale School of Medicine, New Haven, Connecticut, United States; f Université Paris Cité, Paris, France

**Keywords:** functional connectivity, preterm infants, glycemia, diffuse optical tomography, near infrared spectroscopy

## Abstract

**Significance:**

Very preterm infants are prone to large fluctuations in their blood glucose concentration (BGC), i.e., they can experience episodes of hyper- and hypoglycemia, due to impaired glucose control. To date, the relationship among how specific regions of the brain respond to glycemic events has not been fully explored, and characterizing how glucose fluctuations affect region-specific functional connectivity at birth may provide insight into neurodevelopment and could help identify early biomarkers of brain vulnerability in very preterm infants.

**Aim:**

The aim is to evaluate whether the differences in task-free functional connectivity (tfFC) patterns before and after experiencing several days of BGC fluctuations were correlated with changes in the glucose profile during this time interval.

**Approach:**

We continuously monitored both glucose concentration with a continuous glucose monitoring device and brain hemodynamics with diffuse optical tomography in a group of very preterm newborns to conduct tfFC analysis (N=12).

**Results:**

Changes in tfFC patterns between the left frontal and left parietal regions were found to be correlated with the standard deviation of the glucose profile, whereas changes between the central prefrontal cortex and the right prefrontal region were found to be correlated with the maximum value of glucose concentration.

**Conclusions:**

We suggest that changes in the coupling of these brain areas during rest are dependent on and occur during exposures to glycemic changes in the preterm brain.

## 1 Introduction

Very preterm neonates are those born at less than 32 weeks of gestational age (GA), and it is known that this can introduce a myriad of health complications in the first days of life after birth.^1^ In particular, the focus of this paper is the poor regulation of blood glucose concentration (BGC), which can lead to preterms experiencing hyper-^2^ and hypoglycemia.^3^ Typically, in healthy neonates, BGC remains relatively stable within a range called euglycemia (≥72 and ≤144  mg/dL), with hyperglycemia (>144  mg/dL) and hypoglycemia (<72  mg/dL) being states in which BGC goes above and below euglycemia, respectively.^4^^–^^6^

Many studies have shown that health outcomes may be poorer for neonates experiencing hypoglycemia, such as adverse neurodevelopmental outcome^7^^–^^10^ and changes to the central nervous system;^11^ furthermore, neonatal hypoglycemia can lead to secondary epilepsy in later life.^12^ One study, which followed up a cohort of 24 hypoglycemic patients (age range between the first day of life and 5.5 years, median 8 months of age with the follow-ups conducted among the ages of 1.5 to 9 years, median 4 years of age) found developmental delays, learning and behavioral problems, hyperactivity and attention difficulties, autistic features, microcephaly, and cortical blindness across the cohort.^13^ In particular, their structural magnetic resonance imaging (MRI) analysis found in several patients a bilateral volume loss or gliosis in the occipital or parieto-occipital areas. However, it is unclear whether there is a direct and causal link between neonatal hypoglycemia and neurodevelopmental outcome because a study that tracked 745 neonates, who were monitored for glucose concentration at birth, found no significant differences in cognitive or academic skills between the control and affected groups across 3 to 18 years of age.^14^ In addition, another study looking at the neurodevelopmental outcomes at 7 and 9 years of age respectively of a cohort of moderate to severe neonatal hypoglycemic patients found no significant changes in overall cognitive function.^15^

There is also evidence that hyperglycemia in neonates presents adverse health outcomes. For example, in a group of 69 very preterm infants, of which ∼50% experienced hyperglycemia, 13 of them developed neurodevelopmental impairment.^16^ In addition to this, the study found that hyperglycemia directly correlated with lower white matter volume but not with other regional brain volumes. A recent review that included six studies of neonatal hyperglycemia and its effect on neurodevelopmental outcome, over an aggregate of 2226 infants, found that there was an association between hyperglycemia and neurological delay in the first 2 years of life.^17^ They found that, specifically, there were delays in motor function, and this carried on into later childhood. However, their review concluded that across the six studies, the quality of the evidence was “poor,” due to several limiting factors such as sample size, the non-adaption of standardized neurodevelopmental assessment, and the variability of the definition of hyperglycemia.

The existing evidence suggests that both neonatal hypoglycemia and hyperglycemia may be associated with adverse neurodevelopmental outcomes and alterations in the brain structure; however, the nature and strength of these associations remain unclear. Although several studies link later cognitive, behavioral, and neuro-anatomical abnormalities, larger longitudinal investigations do not consistently demonstrate significant long-term impairments, particularly in global cognitive function. Moreover, for hyperglycemia, conclusions are limited by small sample sizes and non-standardized outcome measures, resulting in lower-quality evidence. So, although hypo- and hyperglycemia appear to represent a potential risk factor for the developing preterm brain, there is currently no consensus on causality, critical thresholds, or mechanisms, highlighting the need for studies that directly probe how glucose variability may influence early brain function.

Resting-state functional connectivity (rsFC) is a measure of how spontaneous fluctuations in brain activity relate to each other over time across different spatial areas of the brain.^18^ Multiple studies have demonstrated that it may be a neuromarker for good brain health and function. For example, a study using functional MRI (fMRI) found that rsFC is a neuromarker for sustained attention in children and adolescents:^19^ stronger functional brain networks among motor cortex, occipital lobes, and the cerebellum predicted better performance during a sustained attention task, whereas stronger FC between temporal and parietal regions, as well as intratemporal and intracerebellar connections, predicted a worse performance during the same task.

rsFC has also been evaluated in neonates and infants. For example, Canini and colleagues^20^ used fMRI to calculate perinatal FC (p-FC) markers of prematurity in premature infants, which predicted neurodevelopmental outcomes at 6, 12, 24, and 36 months. These p-FC markers predicted cognitive (p-FC between the bilateral visual cortices at 12 months and p-FC between somatosensory motor and higher-order control cortices at 36 months), language (p-FC between somatosensory motor and higher-order control cortices at 24 months), and socio-emotional (p-FC among the bilateral cerebellar hemispheres at 6 months and p-FC among cortices related to language and emotional control at 12 months) outcomes when compared with term newborns.

In addition, another study used fMRI in very low-birth-weight premature infants and found that by 36 months of age, infants born prematurely had weaker functional connectivity between the visual and motor networks and motor and frontal regions compared with term infants of the same age.^21^ Another study^22^ computed rsFC in adults born preterm and found that there were alterations in emotional processing, represented by changes in functional connectivity between the amygdala and the parietal and temporal cortices, when compared with age-matched controls. They also controlled for sex and socio-economic status (SES), finding no statistically significant differences between males and females and SES categories. All of these studies report promising results on the role of rsFC in the study of brain function and development in preterm infants and its potential as a neuromarker.

There are no studies about whether hypo- or hyperglycemia alters rsFC in full-term infants, or in adults, because no hypo- or hyperglycemic events are expected in these populations. However, it is interesting to highlight what happens in type 1 or 2 diabetic patients, which share the same glucose fluctuation as preterm newborns. Previous studies have investigated how glycemic changes due to diabetes affect rsFC. For example, a meta-analysis of resting-state fMRI studies in type 2 diabetes mellitus (T2DM) patients found hyper- and hypo-activity in patterns of regional spontaneous neural activity across several brain regions.^23^ Further studies found that T2DM affects resting-state attentional networks, which could be related to reduced attention and the hyperglycemic state,^24^ in addition to causing impairments in functional coordination among homotopic brain regions.^25^ Finally, a study investigating type 1 DM (T1DM) in children found that T1DM affected functional activity, namely, a decreased FC of the right inferior temporal gyrus and the right posterior cingulate cortex.^26^ These studies provide a theoretical motivation to investigate how hypo- or hyperglycemia alters rsFC and strengthen the hypothesis that there might be a relationship between adverse glycemic activity and changes in rsFC, also in the preterm population.

So far, all presented studies measured rsFC using fMRI. However, performing fMRI is challenging in awake infants and is not available at the cot-side, and brain activity cannot be continuously monitored for several hours. Possible alternatives to fMRI, aiming to overcome the abovementioned challenges, could be using electroencephalography (EEG), magnetoencephalography (MEG), positron emission tomography (PET), functional ultrasound (fUS), or optical techniques, which have been highlighted in a recent review.^27^

fUS has been shown to be able to measure brain functional connectivity in human neonates at the bedside.^28^ However, it does not currently allow a wide coverage of the cortical surface, therefore limiting its usability to study whole-head functional connectivity. Optical techniques, particularly diffuse optical tomography (DOT),^18^ have been shown to be able to estimate rsFC with a wider cortical coverage and at the cot-side. DOT is a neuroimaging technique that uses changes in the measured intensity of near infrared light^29^ on the surface of the scalp to infer changes in oxygenated (HbO) and deoxygenated (HbR) hemoglobin occurring in the underlying cortical layer. DOT allows 3D images of these changes to be reconstructed on the cortical surface.^30^^,^^31^

DOT is an ideal neuroimaging technique for neonatal and infant imaging because it is non-invasive, non-ionizing, can be portable, and therefore used in challenging environments such as in the neonatal intensive care unit (NICU). Furthermore, compared with other techniques, DOT can be safely and easily used for continuous long-term acquisitions, up to several days.^32^

Several studies estimated rsFC using DOT in several settings and demonstrated its reliability, compared with fMRI rsFC patterns.^18^^,^^31^ rsFC was estimated using fNIRS and DOT also in infants and newborns. For example, the development in global cortical networks in early infancy,^33^ from newborn infants to 3 and 6 months of age, was reported. They showed that rsFC increased as the newborn aged up to 6 months, including an increase in bilateral connections among temporal, parietal, and occipital areas but a decrease in rsFC in the frontal area over time. Another recent study^34^ with fNIRS showed that preterm infants had significantly higher rsFC in both sleep states compared with term-born infants. Such connections may be excessively strong in preterm infants and could potentially lead to atypical neurodevelopmental outcomes. Sleep states in newborns were investigated using rsFC in an HD-DOT study,^35^ and it was shown that quiet sleep had significant differences between the strength of short-range connections to all other types of connections and that the predominance of short-range functional connectivity was not observed for active sleep. rsFC was investigated also in neonates with hypoxic-ischemic brain damage (HIBD).^36^ Newborns with HIBD showed decreased brain functional connectivity when compared with healthy controls, in particular, severe losses of long-range functional connectivity of the contralateral parietal-temporal lobe, contralateral parietal-frontal lobe, and contralateral parietal lobe. All of these cited studies using fNIRS or DOT for measuring rsFC demonstrated that rsFC could be an interesting neuromarker for typical brain function and a predictor of neurodevelopmental outcome.

Past studies investigating the neonatal brain’s response to hypo- or hyperglycemia^37^^,^^38^ found that there was a compensatory increase in cerebral blood flow (CBF) in response to hypoglycemia, and that 30 min after intravenous glucose treatment in hypoglycemic neonates, CBF increased compared with control neonates. Another study investigated how cerebral blood volume (CBV) changed in response to treatment with an intravenous bolus of glucose, using a four-channel NIRS device.^39^ Initially, after the infusion ended, CBV decreased and then reached a steady state 3 min after. Furthermore, there was an inverse relationship between the reduction of CBV and the pre-treatment BGC. A recent study, using a single-channel NIRS device, assessed the relationship between BGC on cerebral regional oxygen saturation (${\mathrm{crSO}}_{2}$) and cerebral fractional tissue oxygen extraction (cFTOE) in full-term and preterm neonates 15 min after birth.^40^ ${\mathrm{crSO}}_{2}$ and cFTOE correlated significantly with BGCs in neonates born at term and preterm, whereby higher BGC’s led to decreases in ${\mathrm{crSO}}_{2}$ and increases in cFTOE. Finally, a recent study from the BabyGlucoLight clinical trial showed that there were spatially dependent correlations between brain hemodynamics and BGC profiles in mild and severe hypoglycemia, respectively, in preterm neonates.^41^ The studies presented above, however, did not measure rsFC because only limited spatial coverage was available or limited acquisition time was possible (e.g., with PET).

From the existing literature on the adverse health outcomes resulting from neonatal hyper and hypoglycemia, in particular, the effects on the preterm neonatal brain, it is clear that there is still a lack of consensus on the direct link between severe glucose fluctuations and neonatal brain modifications. Studies measuring both glucose concentrations and brain hemodynamics in newborns seem to point to a potential influence of glucose values and their fluctuations on brain hemodynamics and therefore to a potential impact of the glucose profile during the first weeks after birth on the developmental patterns of rsFC.

In this study, we aim to further investigate whether the connections among regions in the neonatal brain might be influenced by glucose changes by measuring changes in the pattern of rsFC before and after the newborn experienced episodes of hyper- and hypoglycemia. As sleep states were not monitored in this study, we will call this measure “task-free” functional connectivity (tfFC) instead of rsFC. To achieve this, preterm newborns were monitored with DOT to estimate the tfFC in two separate time windows, one at the beginning of the acquisition and one after several days of acquisition, and continuous glucose monitoring (CGM) to monitor glucose concentration between these two time windows. To the best of our knowledge, no studies have considered how tfFC changes as a function of glycemic variability in preterm neonates, which is the primary aim of this study. Understanding whether and how glucose variability is influencing tfFC is essential to evaluate whether tfFC could be used as a neuromarker to predict how glycemic disregulation could impact neurodevelopment in each baby since the first weeks of life.

## 2 Methods

### 2.1 Patient Recruitment

This study is part of the BabyGlucoLight clinical trial (NCT04347590), which aims to study the relationship between brain hemodynamics and glucose variability (e.g., hypo- and hyperglycemic changes) at birth in very preterm infants, using CGM and DOT. In addition to this, patients were followed up at 1 and 2 years of corrected age to assess neurodevelopmental outcomes and their relationship with glucose variability and brain hemodynamics at birth. The trial was approved by the Institutional Ethics Committee of the University Hospital of Padua (Italy) (4773/AO/19 AOP1813 and 5005/AO/21 AOP2216) and conducted according to the Declaration of Helsinki. Patients were enrolled between March 2020 and June 2023 in the NICU of the Padova University Hospital. Written informed parental consent was obtained before recruitment. There was a prolonged enrollment window due to restrictions in place due to the COVID-19 pandemic in Italy.

Recruitment criteria were GA ≤32 weeks or BW ≤1500 and ≥500  g and availability for monitoring within the first 48 h from birth. Neonates were excluded if they had congenital malformations, perinatal maternal infections, and albinism. In total, 60 patients were recruited, with CGM and DOT data successfully collected in 52 patients (8 patients had problems with the CGM device, and no CGM data were recorded).

### 2.2 CGM Acquisition

CGM was performed using a Medtronic Guardian 3 (United States) device placed on the lateral side of the thigh after disinfection. This device measures an estimate of BGC [sensor glucose concentration (SGC)]. The sampling rate of the SGC was every 5 min, and acquisitions of SGC were performed continuously over several days, up to a maximum of 7 days, with the device being calibrated as per the manufacturer’s instructions. In some instances, there were gaps in the measured glucose data due to the device needing to be recalibrated or disturbances due to clinical intervention. Appropriate exclusion criteria of these gaps will be discussed in Sec. 2.4. From the SGC signal, glycemic events were classified as being severe (S) or mild (m) hypoglycemia or S or m-hyperglycemia. The following SGC thresholds were used to classify events:^4^^–^^6^ >180  mg/dL (S-hyper), >144 and <179  mg/dL (m-hyper), >48 and <72  mg/dL (m-hypo), and <47  mg/dL (S-hypo), with euglycemia being each time point when SGC was ≥72 and ≤144  mg/dL. For an event to be classified, it needed to have at least four consecutive time samples (15 min) within the aforementioned event threshold.

Typical testing, as per clinical standard, for hypo- and hyperglycemia in preterm neonates is usually performed twice a day, with heel prick sampling. This study, instead, uses continuous glucose monitoring performed every 5 min, which means that glycemic events can actually be recorded and monitored as they occur, which is one of the experimental advancements of the study, and allows a more precise evaluation of glucose fluctuations during the first days of life.

### 2.3 DOT Acquisition

DOT data were acquired using an NTS system (Gowerlabs, United Kingdom), which is a continuous wave fNIRS device, with 8 sources and 8 detectors, for up to 64 measurement channels, measuring at 2 wavelengths of 780 and 850 nm with a sampling frequency of 10 Hz. The average head circumference of the patients was 27.9 cm (which equates to a diameter of 8.8 cm if the head circumference was modeled as a circle), which means that the average dimension of the preterm head is approximately the size of an adult fist. This means that, although having only eight sources and eight detectors, these can cover the surface of the cortex with a relatively high-density set of measurements, with different source–detector separation distances sampled.

**Fig. 1:**
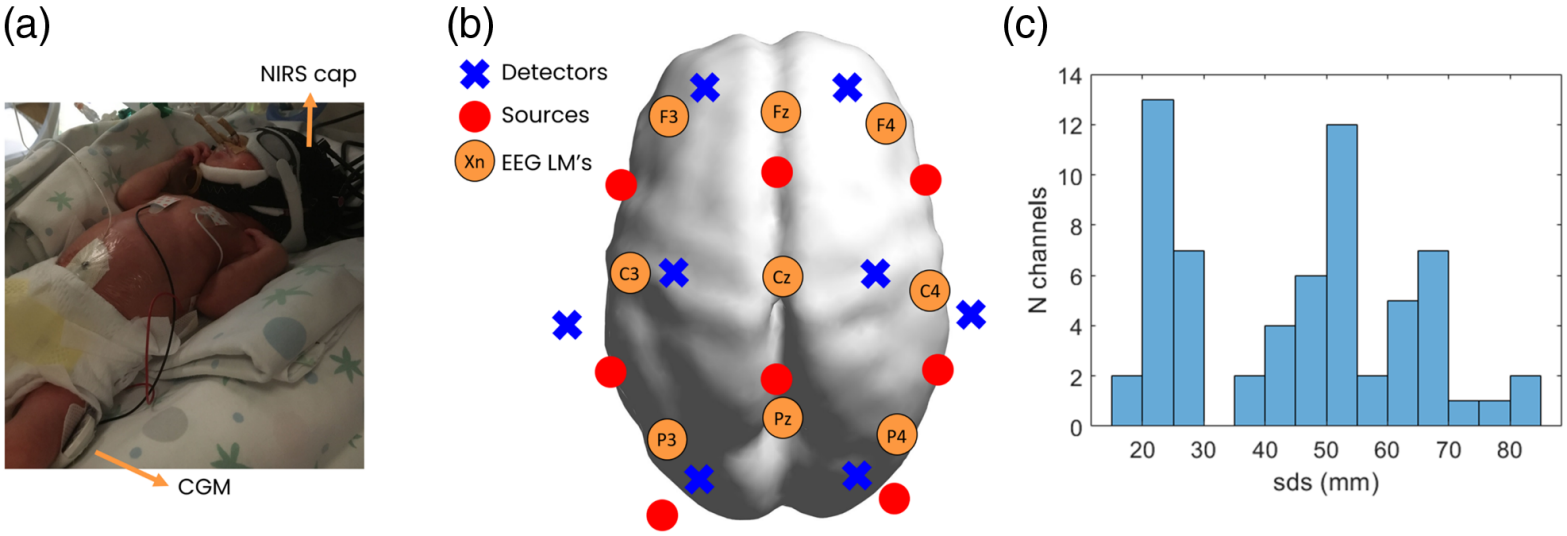
Experimental setup of CGM and DOT acquisition in the NICU. (a) Exemplary preterm neonate with the CGM inserted at the thigh and the DOT cap. (b) Source (in red) and detector (in blue) locations, with annotations of the main EEG 10 to 20 landmark positions for reference. The detectors are approximately placed on F1, F2, CCP5, C1, C2, CCP6, P1, and P2, and the sources are approximately placed on FC3, FCz, FC4, CP3, CPz, CP4, PO5h, and PO6h. (c) Histogram showing the source detector separations of all measurement channels.

The DOT data were collected by the clinicians in the hospital, whereby the NIRS cap was placed on each preterm, and an automatic source calibration took place before data acquisition. The DOT data were acquired simultaneously with the CGM; therefore, data were also acquired continuously for several days, which was found to be safe in a prior study.^32^ The acquisition could be stopped and restarted later on if the preterm had to be manipulated or moved for clinical care. The mean time of the initiation of monitoring was 2 days, 5 h, and 45 min (see Table S1 in the Supplementary Material for details of each patient). The setup can be seen in Fig. 1(a). The positions of the sources and detectors were designed to cover a broad area of the cortex and were placed on the EEG 10-5 landmark positions [Fig. 1(b)]. Sleep states were not monitored during the CGM or DOT acquisition.

### 2.4 Patient Selection Criteria

Of the 60 recruited patients, 52 of them had both CGM and continuous DOT data recorded. Of these, several inclusion and exclusion criteria were defined to have a reliable sample for the tfFC analysis. From the CGM data, mild and severe hypo- and hyperglycemic events were classified for each patient. Concerning CGM data, three inclusion criteria were defined for patient inclusion: (1) the patient’s SGC signal must start in euglycemia, to ensure that, to the best of our knowledge, the first time window for DOT tfFC analysis was not preceded by any glycemic episodes; (2) if gaps were present in the SGC signal, they had to be shorter than a critical gap (45 min), defined by taking the first quartile from the distribution of the duration of all glycemic events across all patients; this rule was made to avoid bias in the computation of the metrics on the SGC signal (e.g., its standard deviation) because the likelihood that the unknown SGC signal was below/above the hypo/hyperglycemic threshold is low within the critical gap; and (3) both SGC and DOT should be available during the time windows used to compute tfFC (CGM and DOT were not always started exactly at the same time). Using these 3 inclusion criteria, out of the 52 patients with both CGM and continuous DOT data acquired, 12 passed all three rules and were selected for tfFC analysis (37 patients passed rule 1, 18 patients passed rule 2, and 48 patients passed rule 3), and these 12 patients will be referred to as the tfFC subgroup. Patient information is reported in Table 1.

**Table 1:** Clinical information of the whole patient group (N=52) and the tfFC subgroup (N=12). Values are expressed as median (iqr 1 − iqr 3) or n (%). Sex is expressed as n male: n female.

	All patients (N=52)	tfFC subgroup (N=12)
Neonates		
Gestational age (weeks)	30 (29 to 31)	31 (30 to 31)
Birth wt (g)	1300 (1040 to 1485)	1390 (1090 to 1510)
Small for gestational age, n (%)	7 (13%)	4 (33%)
Twins, n (%)	16 (31%)	3 (25%)
Sex (male:female)	26:26	6:6
CRIB score	1 (0 to 3)	2 (1 to 3)
IVH grade 3 or 4	4 (8%)	0 (0%)
Mothers		
Maternal diabetes, n (%)	13 (25%)	3 (25%)
PPROM, n (%)	14 (27%)	1 (8%)

CRIB, clinical risk index for babies; IVH, intraventricular hemorrhage; PPROM, preterm prelabor rupture of membranes.

### 2.5 Time Window Selection

tfFC was calculated in two 5-min windows, with the patient in euglycemia, one at the beginning of the acquisition and the other one after a few days. To have reliable estimates of tfFC with the least motion-contaminated DOT data, these 5-min windows were selected within a larger time range, i.e., the time the patient spent in euglycemia at the beginning and at the end of the acquisition. In particular, the first time window started at the beginning of the DOT acquisition because one of the inclusion criteria was that the SGC signal had to start in euglycemia. The end of the first time window was selected as the starting point of the first hyper- or hypoglycemic event of the patient. The last time window, instead, started from the first time sample after the patient’s last full hyper- or hypoglycemic event and ended at either the end of the SGC signal or at the last time sample of SGC in euglycemia, if the acquisition ended outside the euglycemic range. It is worth noting that each patient had their own unique length of first and last time windows, based upon their SGC signal. To homogenize window selection as much as possible, while keeping individual variability, the gap between the end of the first time window and the start of the last time window was calculated for each patient in the tfFC subgroup, resulting in a mean value of 3975 min or ∼2.75 days. Three patients had very small gaps with 510, 115, and 75 min due to only small variability in the glucose trace during the acquisition. In order for there to be an appreciable difference between the first and last time windows, either the end of the first time window was anticipated or the start of the last time window was postponed. By doing this, the gap between the end of the first time window and the start of the last one had a minimum of 510 min, which was originally the third shortest time gap. The mean length of the first and last euglycemic time windows was 1071 and 490 min, respectively, with further information about this for each patient in the subgroup found in Table S2 in the Supplementary Material.

To maximize the quality of the DOT data used for tfFC analysis, an algorithm was implemented to automatically find the best 5-min window within the first and last larger time windows. The selection criteria included having a compromise between the highest number of good channels available and a homogeneous distribution of those channels over the cortex.

A rolling 5-min time window, with increments of 1 min, was used across the entire first and last euglycemic windows. First, the RemoveNoisyChannel function was used from the toolbox Homer2,^42^ with the parameters dRange = [5e−4 3] and SNRrange = 1 to remove any channels with too much noise or signal scarcity/saturation. Then, spectral analysis was performed on each 5-min time window; channels exhibiting peaks with a power spectral density (PSD) larger than a given threshold in the frequency bands of 0.05 to 2 Hz (PSD ≥0.09  W/Hz) or 3 to 4 Hz (PSD ≥0.01  W/Hz) were considered bad channels to be removed due to some light disturbances in the NICU that some time entered in a few detectors.

To evaluate the homogeneity of channel distribution, the sensitivity map of the full array was computed on a mesh model with a GA that corresponded to each patient,^43^ i.e., if the patient had a GA of 31 weeks, then a 31-week neonatal model would be selected. For each time window, the sensitivity map was recomputed using only the available good channels. The average sensitivity value within 9 regions of interest (ROI) computed as 15-mm-radius spheres centered at F3, FZ, F4, C3, CZ, C4, P3, PZ, and P4 10 to 20 positions was computed. The 5-min time window that yielded the highest total sensitivity across the nine ROIs, with each ROI meeting a minimum sensitivity coverage threshold^44^ ($C_{\text{thresh}}$), was considered the best 5-min time window. $C_{\text{thresh}}$ was calculated based upon Eq. (1)

Cthresh=log(100+pthresh100)actvolV^×Δμa, (1)

where $p_{\text{thresh}}$ is the minimum change in the intensity of the signal that can be measured (1%), ${\mathrm{act}}_{\mathrm{vol}}$ is the approximate volume over which a hemodynamic response can be expected to occur ($10\;{\mathrm{mm}}^{3}$), $\hat{V}$ is the median Voronoi volume across nodes of the GM mesh ($\approx 1\;{\mathrm{cm}}^{3}$), and $\Delta \mu_{a}$ is the approximate change in absorption coefficient expected during a hemodynamic response (∼10% of $\mu_{a}\approx 0.001\;{\mathrm{mm}}^{-1}$).

### 2.6 DOT Preprocessing and Image Reconstruction

After the best 5-min windows of DOT data were extracted for the first and last time windows, they were processed for tfFC analysis. Intensity values were converted to optical density, and motion artifact correction was applied, due to the short duration of the time window, to avoid losing other data. As the best quality 5-min windows of data were chosen, and few spike-like motion artifacts were present, wavelet filtering was applied (iqr = 1.2).^45^ Then, bandpass filtering was applied with cut-off frequencies at 0.009 and 0.08 Hz.^46^ The processed optical density data were then fed to the image reconstruction pipeline.

**Fig. 2:**
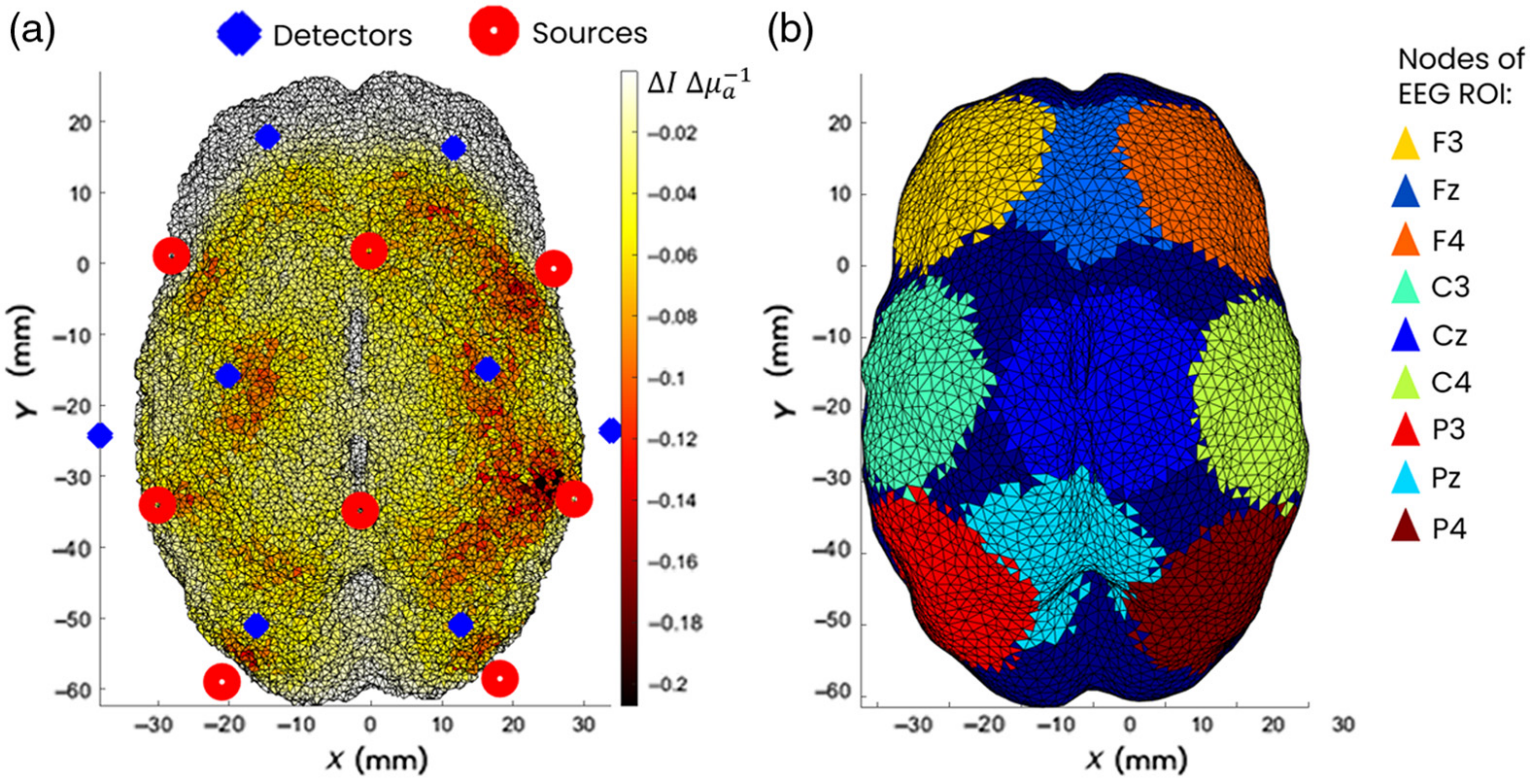
(a) Example Jacobian based upon good channels, for patient 25 (GA model was 31 weeks), during the best 5-min window for the first tfFC analysis. The coverage threshold was 0.0082 $\Delta I\Delta \mu_{a}^{-1}$. (b) Nine EEG ROIs for this patient.

For each patient, the 3D neonatal mesh model^43^ corresponding to the nearest GA of the patient was selected. NIRFAST^47^ was used to calculate the Jacobian at both wavelengths for each mesh model used with optical properties taken from Uchitel and colleagues^35^ (Table S1 in their Supplementary Materials, 735 and 850 nm). An example Jacobian can be seen in Fig. 2(a).

Image reconstruction was performed by solving the inverse problem using Tikhonov regularization with a regularization parameter of 0.1.^48^ The pseudoinversion of the Jacobian, $J^{\#}$, was calculated as reported in Eq. (2)

$$J^{\#}=\frac{J^{\prime }}{\mathrm{JJT}+I\times \lambda \times \max(S)},$$ (2)

where $\mathrm{JJT}=J\times J^{\prime }$, I is the identity matrix, λ is the regularization parameter, and max(S) is the maximum value of the singular value decomposition of JJT. Changes in HbO and HbR were then calculated for each node of the volumetric mesh and then mapped to the gray matter smoothed surface mesh by assigning to each node of the cortical surface mesh the mean value of the volumetric ones present within a 3-mm-radius sphere centered on that node.

### 2.7 tfFC Analysis

tfFC was calculated among nine ROIs, defined as spheres of 15-mm radius centered on 9 EEG 10 to 20 positions: F3, FZ, F4, C3, CZ, C4, P3, PZ, and P4, which can be seen in Fig. 2(b). Analysis was performed in ROI space as a way to compare broad areas of the preterm brain because using smaller parcellations of specific brain regions would be too spatially specific, given the uncertainties from the inherent resolution of the DOT and from small discrepancies in cap placement among patients. Spheres of radius 15 mm were used because these created the largest ROIs with minimal spatial overlap in the frontal to parietal direction. Each node had a unique label; if a node belonged to both the central and lateral ROIs, it was assigned to the lateral one, so there were no duplicate nodes used for different ROIs. To ensure reliable coverage of the individual DOT data in each ROI, for each patient, only nodes within the ROI, with a sensitivity above a coverage threshold, computed as described in Brigadoi et al.,^44^ were considered to estimate the average ROI HbO and HbR signal. If a given ROI had less than one-third of its nodes above the coverage sensitivity, that ROI was not considered for tfFC analysis in that patient. All patients had all ROIs included, apart from patient 14 which had ROI F3 and Fz excluded.

tfFC was calculated, for both HbO and HbR, by computing the Pearson correlation coefficient (R) between each pair of ROIs, for the first and last 5-min time windows, separately. R values of the correlations were then transformed into Fisher Z-scores. Then, a two-sample t-test was performed on the Fisher Z-scores comparing the tfFC pattern in the first and last time window, for both HbO and HbR separately, to evaluate whether the pattern differed in the two time windows. Furthermore, to evaluate which ROI pair had a significant correlation within the network, t-values were calculated, separately for each 5-min time window, across all patients for each ROI pairing, as shown in Eq. (3)

$$t_{\mathrm{HbXrsFCn}}=\frac{\text{mean}(Z_{\mathrm{HbXrsFCn}})}{{\mathrm{SE}}_{\mathrm{HbXrsFCn}}},$$ (3)

where $t_{\mathrm{HbXrsFCn}}$ is the t-value for the tfFC of HbO or HbR for the first or last time window, $Z_{\mathrm{HbXrsFCn}}$ is the Fisher Z-score, and $SE_{\mathrm{HbXrsFCn}}$ is the standard error, which is calculated as the standard deviation of $Z_{\mathrm{HbXrsFCn}}$ divided by the square root of the number of patients. Then, to account for the multiple comparisons problem, false discovery rate (fdr) correction was applied to the p-values of $t_{\mathrm{HbXrsFCn}}$ and $Z_{\mathrm{HbXrsFCn}}$, using the Benjamini–Hochberg method^49^ (fdr statistical significance was p≤0.05).

### 2.8 Correlation Between tfFC and Glucose Metrics

To evaluate whether glucose changes during the first days of life might be related to changes in tfFC pattern between the first and last time window of DOT analysis, eight metrics were calculated from the SGC signal across the entire CGM acquisition of each patient. These were (1) the mean and (2) standard deviation (SD) of the SGC signal across the entire acquisition, (3) the minimum (MIN) and (4) maximum (MAX) values of the SGC signal considering the whole acquisition, (5) the percentage of time spent within the euglycemic range (TIR %), (6) the percentage of time spent outside the euglycemic range (TOR %), (7) the percentage of time spent below the euglycemic range (TBR %), and (8) the percentage of time spent above the euglycemic range (TAR %).

Correlations were calculated among the changes in tfFC pattern for each ROI pair from the first to the last time window for both HbO and HbR and the glucose metrics defined above.

## 3 Results

### 3.1 Glucose Results

**Fig. 3:**
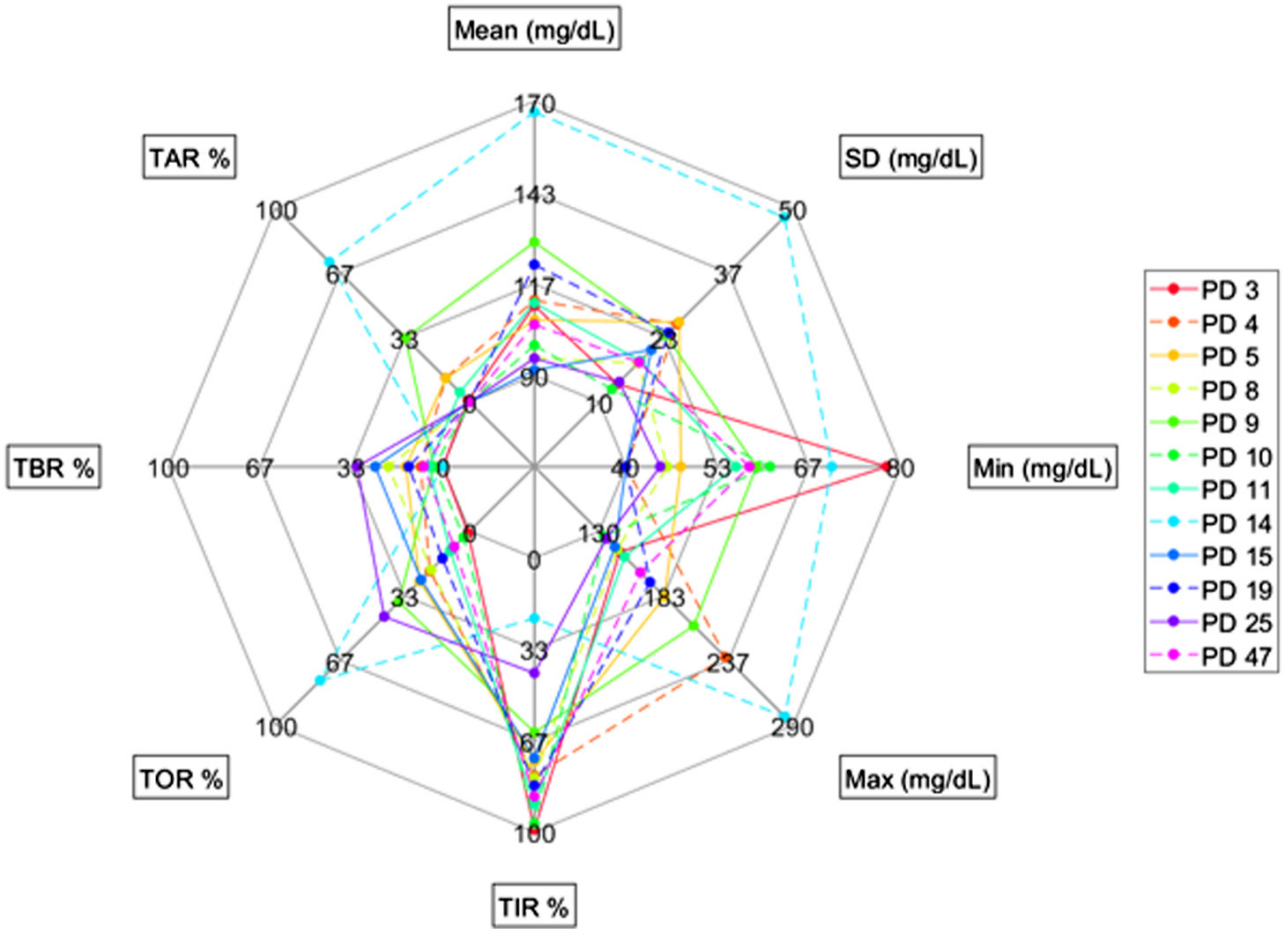
Spider plot of the glucose metrics computed for the 12 patients in the tfFC subgroup. Each color line denotes a patient, and each vertex of the spider plot represents a different metric. TIR %, percentage of time spent within the euglycemic range; TOR %, percentage of time spent outside the euglycemic range; TBR %, percentage of time spent below the euglycemic range; TAR %, percentage of time spent above the euglycemic range.

Figure 3 displays a spider plot showing the estimated glucose metrics for each patient, with Table 2 showing how the mean SGC and time in range (TIR) percentage in this study compared with other similar studies.

**Table 2:** Comparison of the SGC of the tfFC subgroup in this study and those of similar other studies. The first column denotes the study and the specific cohort being referred to, with the number of patients used for that given analysis, N; the second column presents the mean SGC given with the standard deviation or iqr in brackets; and the last column shows the TIR percentage. Blinded and unblinded in Galderisi et al.^7^ refers to two different treatment arms using CGM, one in which CGM was actively used to try to prevent glucose fluctuations (unblinded) and the other one in which CGM measures were blinded to clinicians.

Study (N)	Mean SGC (mg/dL)	% TIR (72 to 144 mg/dL)
tfFC subgroup (12)	111.8 (20.5)	74
Beardsall et al.^5^ CGM (70)	117.1 (21.6)	74
Galderisi et al.^6^ blinded (25)	102.6 (100.8 to 106.2)	68
Galderisi et al.^6^ unblinded (25)	97.2 (93.6 to 100.8)	84

One of the features of the subgroup of 12 newborns selected for the tfFC analysis is the high between-subject variability in glucose metrics (Fig. 3). Some metrics had lower between-subject variability; for example, 9/12 patients spent at least two thirds of the acquisition within the euglycemic range (TIR %), and all 12 patients spent less than one third of the acquisition in hypoglycemia. Some other metrics, instead, had higher between-subject variability, such as mean SGC and standard deviation of SGC. The heterogeneity of glucose profiles within the subgroup, including patients at the extreme ends of glycemic variability, such as the one spending 99% of the time within euglycemia and the other one spending 22% of the time within euglycemia, means that there is a good range of differing glycemic characteristics that changes in tfFC can be evaluated against.

From Table 2, it can be seen that the mean SGC from the tfFC subgroup is similar to that of the other two similar studies^5^^,^^6^ with the percentage of TIR being the same as the CGM cohort from Beardsall and colleagues and in-between the blinded and unblinded CGM TIR from Galderisi and colleagues. An example SGC trace and further information on the number of glycemic events for the tfFC subgroup can be found in Figs. S1 and S2 of the Supplementary Materials, respectively.

### 3.2 tfFC Results

**Fig. 4:**
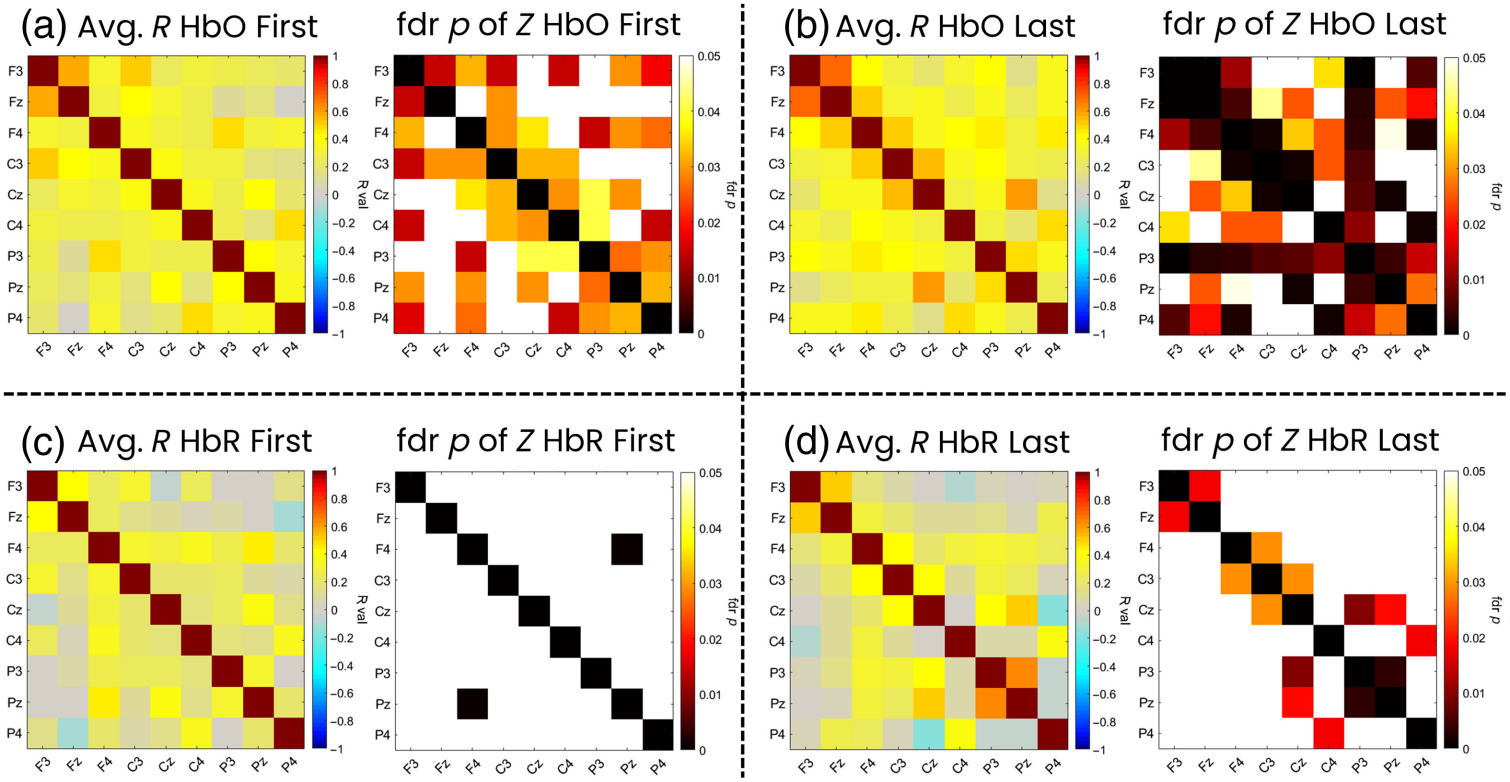
tfFC for HbO and HbR in the first and last 5-min time windows. (a) and (b) On the left, the average R value across all subjects for each ROI pair for HbO in the first time window (a) and last time window (b). The graph on the right shows the fdr corrected p-values of the Z-scores computed across patients. (c) and (d) Same as panels (a) and (b) but for HbR.

Figure S3 in the Supplementary Material displays an example of raw DOT signals in one of the patients during one of the euglycemic windows. An example of a tfFC matrix for one patient can be found in Figs. S4 and S5 of the Supplementary Materials. Figure 4 reports the average R values for each ROI pair for HbO and HbR in the first 5-min time window [panels (a) and (c)] and in the last 5-min time window [panels (b) and (d)], as well as whether the correlation was significant, by reporting the fdr-corrected p-values. In the same figure, the results of the comparison between the tfFC pattern of the first and last 5-min time window are reported.

The average R values for HbO across patients and ROIs in the first and last time windows are respectively 0.37 and 0.43, whereas for HbR, 0.28 and 0.27, which are smaller in magnitude compared with HbO. These global averages provide a measure of the overall strength of network connectivity, indicating stronger connectivity for HbO than HbR and showing a small increase in mean HbO connectivity over time. For the homotopic connections, F3–F4 were 0.34 (HbO first), 0.42 (HbO last), 0.24 (HbR first), and 0.17 (HbR last); C3–C4 were 0.28 (HbO first), 0.35 (HbO last), 0.20 (HbR first), and 0.10 (HbR last); and P3–P4 were 0.31 (HbO first), 0.35 (HbO last), 0.02 (HbR first), and −0.05 (HbR last). For HbO, these all exhibited statistical significance as indicated in Fig. 4, whereas for HbR, none of the homotopic ROI pairs had statistical significance.

When directly comparing the tfFC patterns between the first and last 5-min windows, only one ROI pair (P4–FZ) for HbO and three ROI pairs for HbR (P4–FZ, PZ–P3, and C4–F3) reached statistical significance, although not surviving multiple comparison correction. We report these results as exploratory. In particular, tfFC increased for HbO in P4–Fz, whereas for HbR, and it increased in Pz–P3 and P4–Fz and decreased in C4–F3. Interestingly, one ROI pair (P4–Fz) was common for both chromophores.

However, when considering the ROI pairs that exhibited a statistically significant correlation in the two time windows, a different picture emerged. In particular, 22 ROI pairs achieved statistical significance for HbO in the first time window and 27 in the last one, whereas only 1 ROI pair achieved statistical significance for HbR in the first time window and 7 in the last one. These results might suggest that there is greater variability in tfFC across patients for HbR than HbO, and as discussed by Santosa and colleagues,^50^ the larger number of significant HbO connections could reflect the higher sensitivity and signal amplitude of HbO relative to HbR.

### 3.3 Correlation Between tfFC and Glucose Metrics

**Fig. 5:**
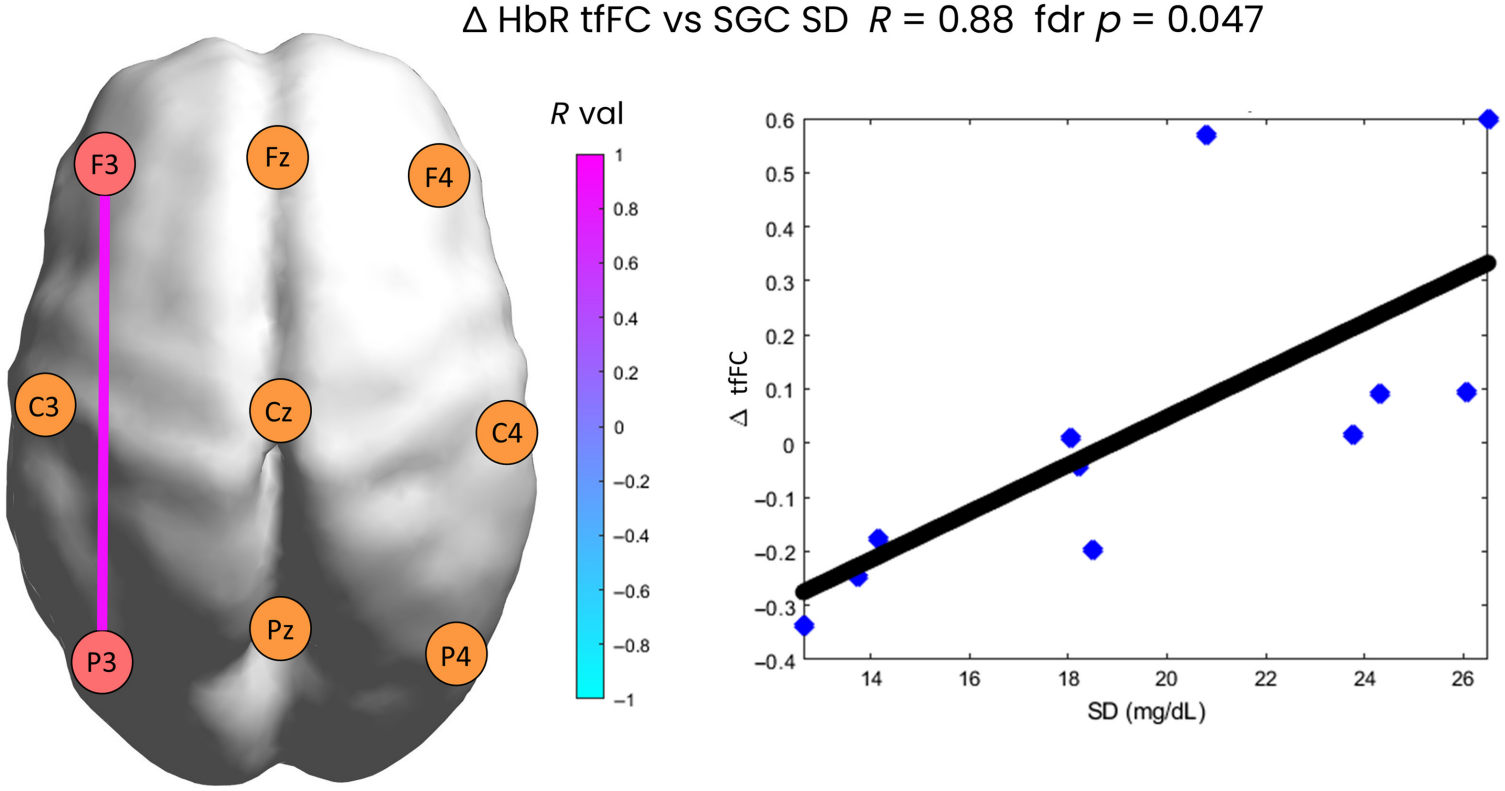
Statistically significant correlation between changes in tfFC for HbR and the SD glucose metric. On the right, a scatter plot showing the correlation is presented. Each dot represents a patient.

**Fig. 6:**
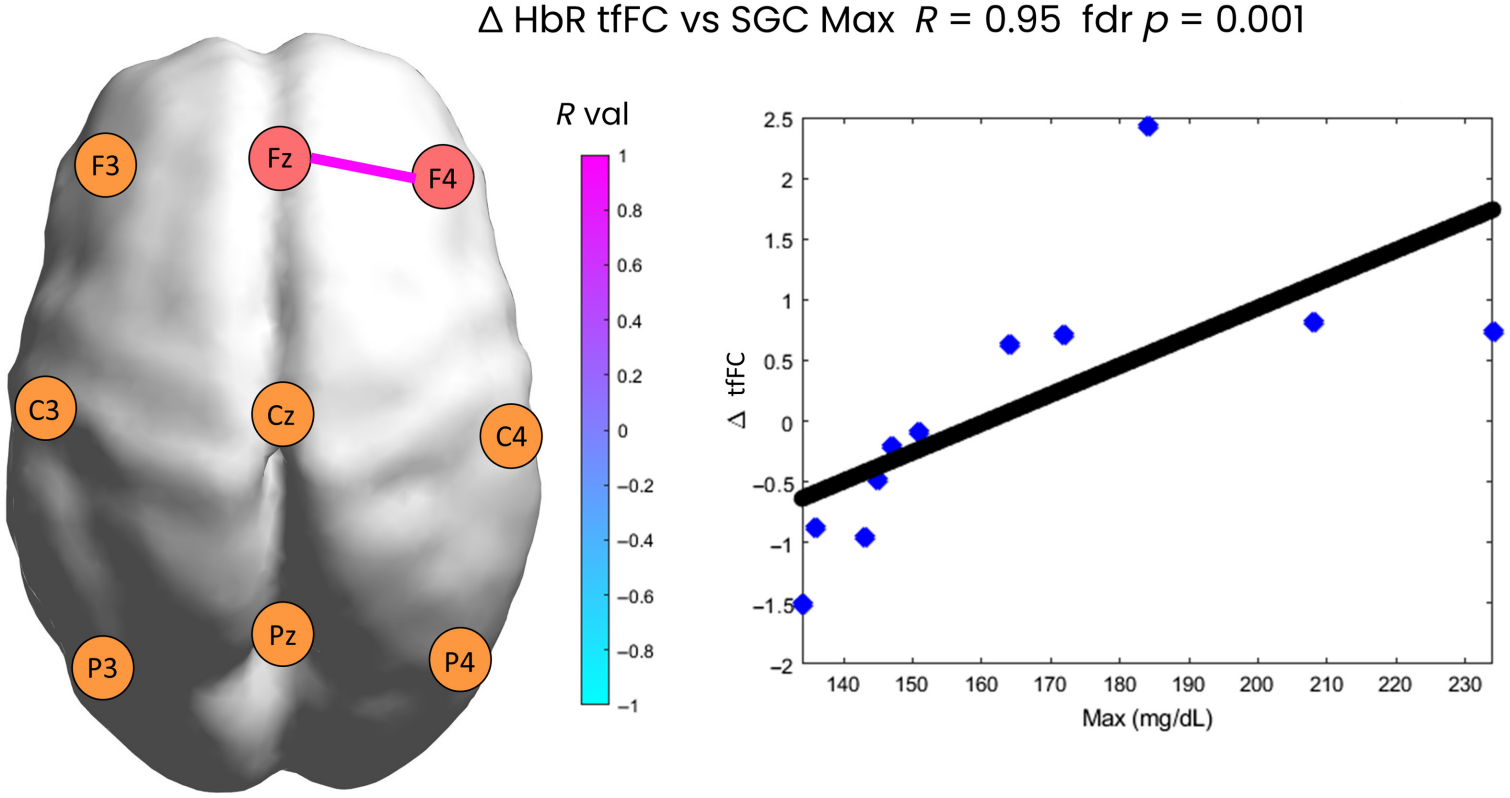
Statistically significant correlation between changes in tfFC for HbR and the MAX glucose metric. On the right, a scatter plot showing the correlation is presented. Each dot represents a patient.

Changes in tfFC for each ROI pair between the first and last 5-min time window, ΔtfFC, were compared with key glucose metrics computed across the entire SGC signal for each patient. The two fdr-corrected statistically significant comparisons that emerged from this analysis are presented in Figs. 5 and 6. Figure 5 shows that for the F3–P3 ROI pair, there is a strong positive correlation (R=0.88) between ΔtfFC in HbR and the standard deviation of the glucose signal. Patients with a stronger tfFC in F3–P3 in the last time window compared with the first one are characterized by a more stable glucose trace (lower SD), whereas patients with a stronger tfFC in the first than the last time window are characterized by stronger fluctuations in glucose trace. In other words, increases in the tfFC between left frontal and left parietal areas of the brain occur in patients with higher variability in the glucose trace, whereas decreases in tfFC among these areas of the brain occur in patients with low variability in glucose concentration over time.

Figure 6 shows that for the Fz–F4 ROI pair, there is a strong positive correlation (R=0.95) between the ΔtfFC in HbR and the maximum of SGC (MAX). Patients with high glucose values (likely having experienced hyperglycemic events) show an increase in tfFC between the first and last time window in the right prefrontal area, whereas patients with lower glucose values (likely having experienced only hypoglycemic events) show a decrease in tfFC between the first and last time window.

## 4 Discussion

This study demonstrated the first use of DOT on preterm infants to evaluate whether tfFC changes during the first days of life are linked to glucose variability and hypo/hyperglycemic events. We successfully collected continuous glucose concentration and DOT data on a cohort of 52 preterm neonates, with 12 of these neonates selected based on our inclusion criteria. Previous studies investigating the relationship between glucose changes and brain hemodynamics could not investigate resting state networks due to limited cortical coverage. Here, the use of DOT is essential because it allows the continuous monitoring of changes in brain hemodynamics across a broad area of the cortex. As previous studies^51^ found that rsFC can act as a neuromarker for brain function and neurodevelopmental outcome, being able to assess rsFC across broad areas of the preterm brain at the cot-side seems mandatory and could facilitate being able to investigate the effects of any clinical event (here, glycemic events) on brain health and neurodevelopmental outcome.

First, we evaluated the tfFC patterns separately for the two time windows to evaluate the differences in their spatial configurations. This analysis identified ROI pairs whose correlations were consistently different from zero at the group level. However, following Santosa and colleagues,^50^ such statistical significance should be interpreted cautiously, given signal-to-noise characteristics and multiple-comparison constraints. A higher number of ROI pairs exhibited statistically significant tfFC in the last time window compared with the first time window (Fig. 4). This was true for both HbO and HbR, although the number of significant tfFC for HbO was much higher than for HbR. In particular, for HbO, there seems to be an increase in regions showing significant functional connectivity from the first to the last time windows. These results, therefore, indicate that stronger tfFC occurs among more areas of the brain as the newborn’s brain is developing. These results might be in line with the hypothesis that the newborn brain is building its organization during the early phases of development,^52^ by increasing the connection among different areas of the brain and pruning the superfluous connections^33^^,^^53^^,^^54^ Alternatively, if these changes were linked to glucose variability, the increased number of spatial regions exhibiting significant functional connectivity could represent a compensatory or deficient response against hyper or hypo-glycemic events.

Second, we evaluated whether tfFC patterns significantly differed in the two time windows of analysis (Fig. 4). No significant differences were found after correcting for multiple comparisons. However, due to the limited sample size, it is worth mentioning the exploratory results that emerged from this analysis before the correction. The fronto-parietal correlation between Fz and P4 showed a significant increase in correlation for both HbO and HbR from the first to the last time window. The other two correlations, Pz–P3 and C4–F3 showed significant changes in tfFC between the first and the last time window but for HbR only. However, none of the changes of tfFC of these ROI pairs from the first to the last time window exhibited statistically significant (FDR-corrected) correlations with any of the SGC metrics. Therefore, it is likely that these changes in tfFC between these ROI pairs might be due to preterm brain maturation or other confounding clinical factors that could occur in these patients in the first days after birth. Furthermore, given the exploratory nature of these analyses, these results should be confirmed using a larger sample size to reach statistical significance.

Although significant changes in tfFC between the two time windows were not correlated with glucose metrics, we did find two significant correlations between glucose metrics and tfFC in other brain areas, demonstrating for the first time a link between glycemic variability and tfFC in the preterm brain. In particular, the change in functional connectivity over time in the left fronto-parietal (F3–P3) connection was significantly correlated with the standard deviation of the glucose concentration (Fig. 5), demonstrating that when the variability of SGC was higher, functional connectivity between the left frontal and left parietal cortex increased over time. This suggests that the neonatal brain might respond to an increase in glycemic variability by increasing the functional connectivity of these areas. A study by He and Parikh^52^ showed that the fronto-parietal network matures significantly in preterm infants from 32 to 39 and 52 weeks (post-menstrual age), which means that an increase in functional connectivity during the first few days from premature birth may be damaging. This is based upon evidence by Shiraki and colleagues,^34^ who found that FC networks may be excessively strong in preterm infants, which potentially led to atypical neurodevelopmental outcomes.

The second statistically significant correlation occurred in the frontal areas, between the Fz–F4 ROIs and the SGC MAX, showing increases in tfFC when experiencing high SGC values. These brain areas are part of the developing executive control network (ECN),^55^ and excessively high increases in tfFC here could be indicative of potential atypical neurodevelopmental outcome, as shown in a previous study investigating rsFC in preterm infants.^34^

In this study, we chose to compare the two euglycemic time windows and correlate their tfFC patterns with glycemic variability instead of comparing tfFC patterns between euglycemic and hypo/hyperglycemic windows. This choice was made because each glycemic event might have a different impact on brain hemodynamics across babies and within the same baby; therefore, changes in tfFC in one hypo/hyperglycemic event may be different from changes in tfFC in another event, making it hard to isolate its effect. Therefore, we decided to look at the overall impact of glycemic variability during a few days, on the changes in tfFC while the baby was in a stable glucose state.

The results presented here are further strengthening the hypothesis of the impact of glucose changes on neurodevelopment and therefore the potential importance of using CGM and controlling glucose variability in clinical settings.

There are a few limitations of note for this study. First, despite a total recruitment of 60 patients, with data collected successfully in 52 of them, only 12 of them were selected for the final tfFC analysis. The exclusion of the other 40 patients was due to conservative exclusion criteria designed to ensure consistency across patients, in particular that the first tfFC time window would be, to the best of our knowledge, before any glycemic events, and no major gaps were present in the glucose signal. A related limitation is that major glycemic events could have occurred within the first 48 h after birth, meaning that the tfFC in the first time window might already be impacted by previous glucose variability. CGM was placed on the baby as soon as possible once the newborn was stable in the NICU after birth and after having parents signed written informed consent.

Another limitation concerns not using subject-specific registration for the source–detector probes. However, this limitation is in part mitigated by the fact that the preterm neonate’s head is incredibly small (diameter on the order of 10 cm) such that the neoprene cap was designed to fit about the ears of the neonate, minimizing variation of cap alignment. Furthermore, the ROIs used for the tfFC analysis were purposely kept to be “broad” across the nine ROIs, so that if there were small differences in the alignment of the cap, the sampling area of a given ROI should eventually be consistent across subjects.

The choice of the length of the tfFC window was made to find a compromise between having a long enough window to compute tfFC reliably^56^ and having a short enough window with high-quality data, given that DOT data were acquired continuously, also during clinical practice and parental visits. However, longer time windows could have improved the reliability of the estimates. Another limitation of the study was the use of ROIs based on 10 to 20 positions. An anatomical parcellation, currently not available with the 4D neonatal model employed, could have provided more specific results. Finally, sleep states were not monitored in this study; therefore, the term “tfFC” was used instead of “rsFC,” and therefore, during the start and end 5-min time windows, FC may be influenced by the sleep state of the preterm. However, the correlations found between changes in tfFC and the glycemic profile were corrected for multiple comparisons, and it is likely that, due to the selection of the specific time for each time window being when the data quality was the best, the preterm was in a quiet sleep state, because this sleep state would exhibit the least amount of movement.

## 5 Conclusion

This study demonstrated the feasibility to measure simultaneously and continuously DOT and CGM data on a cohort of preterm neonates in the NICU and to estimate the cortical tfFC across 9 ROIs on a subgroup of 12 patients. A particularly interesting result that emerged from the tfFC analysis was the correlation, in the left fronto-parietal network, of the changes in tfFC over time and the standard deviation of glucose concentration during the first days after birth, as well as the correlation, in the right frontal network, between the changes in tfFC and the maximum value of glucose concentration. These correlations may indicate a direct impact of glucose variability on brain coupling, either as a compensatory adjustment to these metabolic changes or because these areas form the FPN and ECN networks; this change in coupling could be indicative of a potential neurodevelopmental impairment.

## Supplementary Material

10.1117/1.NPh.13.S1. S13008.s01

## Data Availability

The code used for this study can be found on GitHub at https://github.com/sbrigadoi/BabyGlucoLight in the CODICE%20INVIO1 folders. The data for this study can be found at https://researchdata.cab.unipd.it/id/eprint/1655.
