# Supplementary material for: Task-free functional connectivity changes before and after hyper- and hypoglycemia in very preterm neonates
Source: Neurophotonics. 2026 Mar 31;13(Suppl 1):S13008. doi: 10.1117/1.NPh.13.S1.S13008 (PMC13037429; doi:10.1117/1.NPh.13.S1.S13008)
Supplement: Supplementary file 1 [file NPh_013_S13008_SD001.pdf]

Supplementary materials, by Guy A. Perkins:

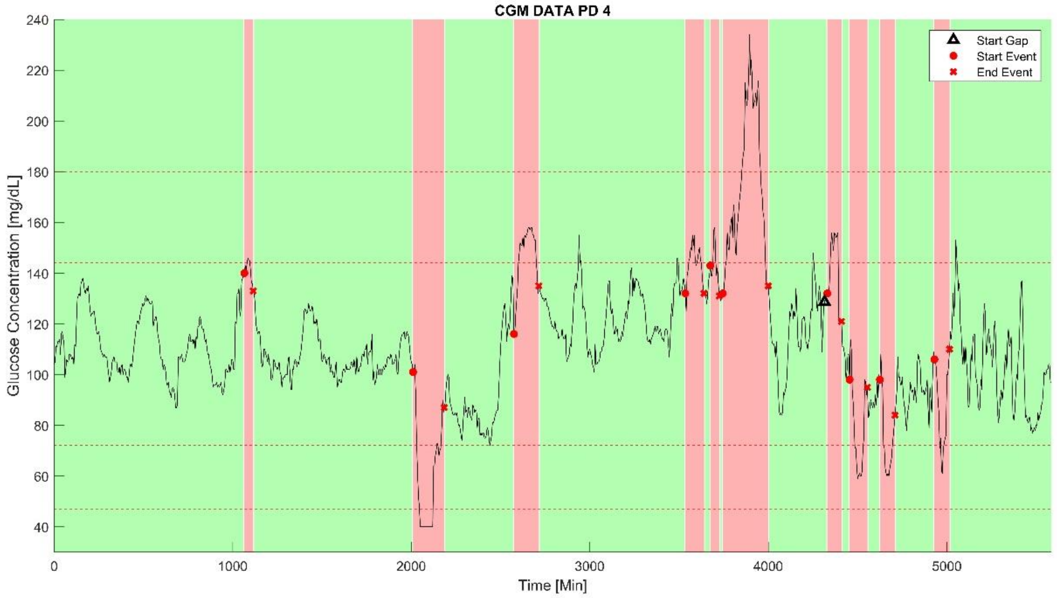


Supplementary figure 1 – Example SGC, shown for PD4. Note that for this specific patient, the SGC gap is only 1 time sample, so only a 'start gap' was recorded.

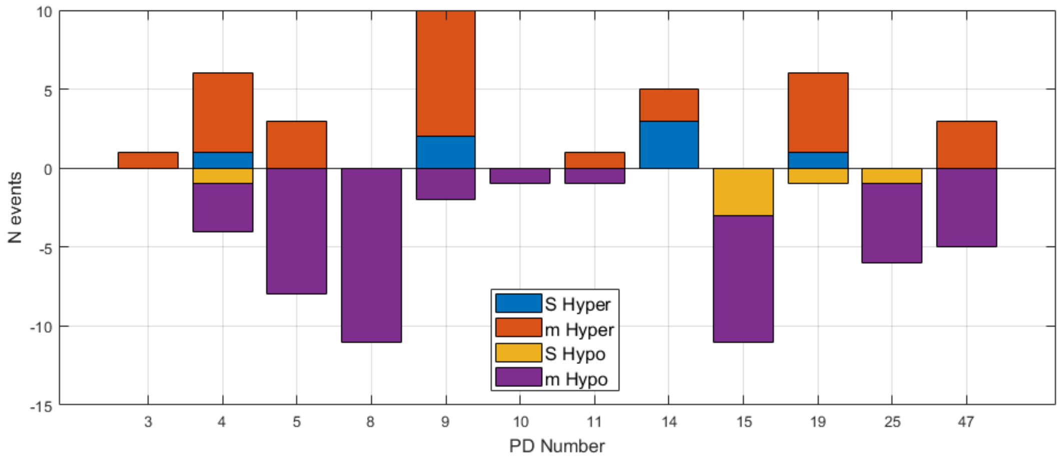


Supplementary figure 2 – The number of glycaemic events for the tfFC subgroup.

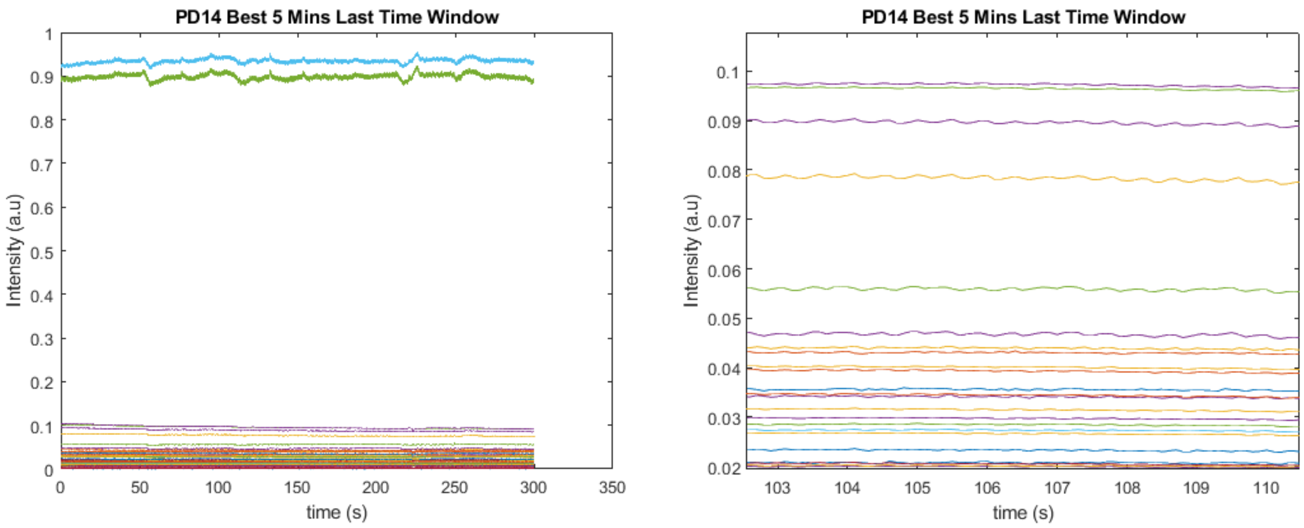


Supplementary figure 3 – Example NIRS data from PD14 for the best 5 minute window for the last euglycemic time window (left entire 5 minutes, right a several second time period during the 5 minute time window, showing the cardiac pulse).

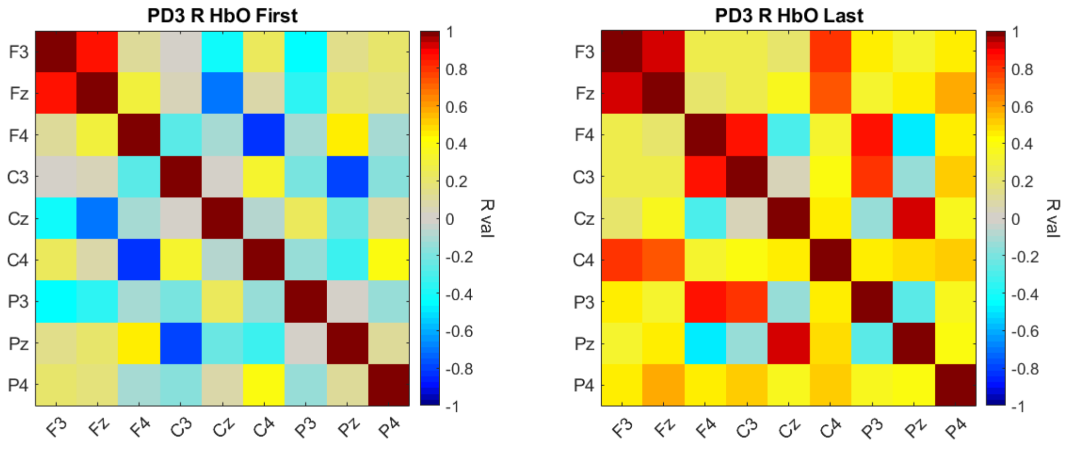


Supplementary figure 4 – Example tfFC matrix for PD4, looking at the first and last time window for HbO.

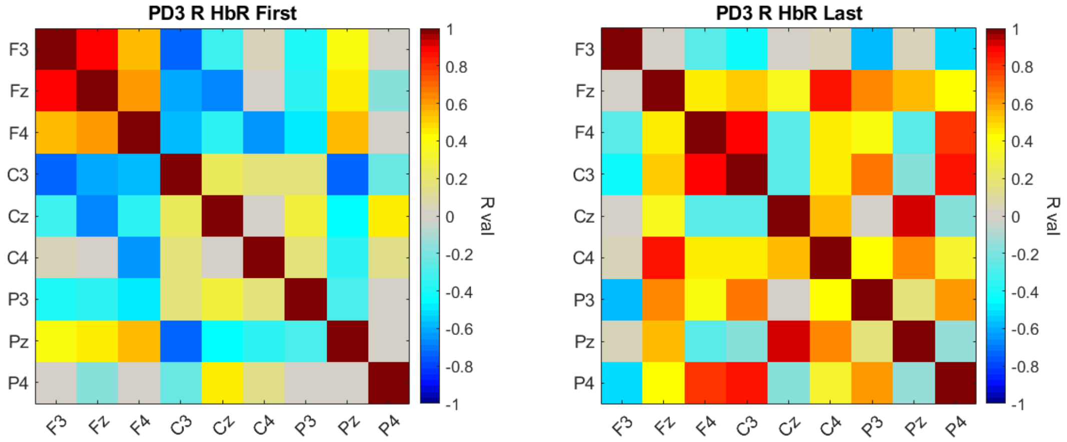


Supplementary figure 5 – Example tfFC matrix for PD4, looking at the first and last time window for HbR.

Supplementary table 1 – The time from birth to initial monitoring each patient in the tfFC subgroup, given in both minutes and hours.

| Patient ID | Time monitoring started since birth (minutes) | Time monitoring started since birth (Hours) |
|---|---|---|
| 3 | 4.876 | 81,3 |
| 4 | 3.787 | 63,1 |
| 5 | 2.641 | 44,0 |
| 8 | 2.298 | 38,3 |
| 9 | 1.367 | 22,8 |
| 10 | 667 | 11,1 |
| 11 | 3.338 | 55,6 |
| 14 | 5.304 | 88,4 |
| 15 | 8.041 | 134,0 |
| 19 | 2.286 | 38,1 |
| 25 | 3.832 | 63,9 |
| 47 | 279 | 4,7 |

Supplementary table 2 – The length of the first and last euglycaemic time windows for each patient in the tfFC subgroup.

| Patient ID | Length Start Euc. window (mins) | Length End Euc. window (mins) |
|---|---|---|
| 3 | 4575 | 1800 |
| 4 | 1060 | 565 |
| 5 | 10 | 135 |
| 8 | 185 | 225 |
| 9 | 125 | 35 |
| 10 | 1750 | 1525 |
| 11 | 205 | 480 |
| 14 | 40 | 195 |
| 15 | 885 | 10 |
| 19 | 1660 | 110 |
| 25 | 150 | 400 |
| 47 | 2215 | 405 |
